# Fermented Vegetables: Health Benefits, Defects, and Current Technological Solutions

**DOI:** 10.3390/foods13010038

**Published:** 2023-12-21

**Authors:** Xiqian Tan, Fangchao Cui, Dangfeng Wang, Xinran Lv, Xuepeng Li, Jianrong Li

**Affiliations:** 1 College of Food Science and Technology, Bohai University, Jinzhou 121013, Chinaxuepengli8234@163.com (X.L.); lijr6491@163.com (J.L.); 2 National & Local Joint Engineering Research Center of Storage, Processing and Safety Control Technology for Fresh Agricultural and Aquatic Products, Jinzhou 121013, China

**Keywords:** fermented vegetables, nutritional composition, function, nitrite, biogenic amine, sterilization, non-thermal technology

## Abstract

This review summarizes current studies on fermented vegetables, analyzing the changes in nutritional components during pickling, the health benefits of fermented vegetables, and their safety concerns. Additionally, the review provides an overview of the applications of emergent non-thermal technologies for addressing these safety concerns during the production and processing of fermented vegetables. It was found that vitamin C would commonly be lost, the soluble protein would degrade into free amino acids, new nutrient compositions would be produced, and the flavor correlated with the chemical changes. These changes would be influenced by the variety/location of raw materials, the original bacterial population, starter cultures, fermentation conditions, seasoning additions, and post-fermentation processing. Consuming fermented vegetables benefits human health, including antibacterial effects, regulating intestinal bacterial populations, and promoting health (anti-cancer effects, anti-diabetes effects, and immune regulation). However, fermented vegetables have chemical and biological safety concerns, such as biogenic amines and the formation of nitrites, as well as the existence of pathogenic microorganisms. To reduce hazardous components and control the quality of fermented vegetables, unique starter cultures, high pressure, ultrasound, cold plasma, photodynamic, and other technologies can be used to solve these problems.

## 1. Introduction

Fermentation is a traditional method used to preserve vegetables. Many regions worldwide have a tradition of consuming fermented vegetables [1,2,3]. Numerous fermented vegetables exist according to the raw materials, formula, and fermentation technologies used. Typical fermented vegetables include sauerkraut [4,5], paocai [6], zhacai [7], and kimchi [8]. The primary constituents for producing fermented vegetables are cruciferous vegetables, such as cabbage, kale, mustard green, or radish. Other prevalent vegetables include chili pepper [9], lotus root [10], carrot [11], ginger [12], cucumber [13], eggplant, beetroot [14], garlic [15], olive [16], papaya, and chayote [17]. There are variations in the production procedures used for different fermented vegetables. However, the primary processing procedure includes the pretreatment of raw materials, brining, and seasoning, and fermenting naturally or under the activity of certain starter cultures, and the fermentation process might last from several days to months. The predominant fermentation techniques are dry salting and brine-pickling [18]. The quality of fermented vegetables is affected by the metabolism of the microbial population (lactic acid bacteria and yeast) during the fermentation process [19], which includes complex microorganisms, especially the autochthonous microbial community [20], the activity of enzymes, the varieties of the seasonings, and the fermentation conditions [21]. 

In addition to enriching consumers’ dietary structure, fermented vegetables can improve human health. Not only do the probiotics in fermented vegetables have specific functional characteristics, but their metabolic activities using plant substrates can also convert active compounds, or the precursors of the active compounds, in plants, which would release or transform into biologically active substances. The nutrients in fermented vegetables include short-chain fatty acids, minerals, polyphenols [22], γ-aminobutyric acid (GABA) [23], B vitamins, flavonoids, and antibacterial peptides [24], which can stimulate appetite, inhibit pathogens [25], regulate intestinal flora [26], avoid obesity [27], pacify emotion [28], prevent cancer [29], treat chronic diseases [30], reduce local inflammation [31], and prevent severe COVID-19 infection [32]. 

**Figure 1:**
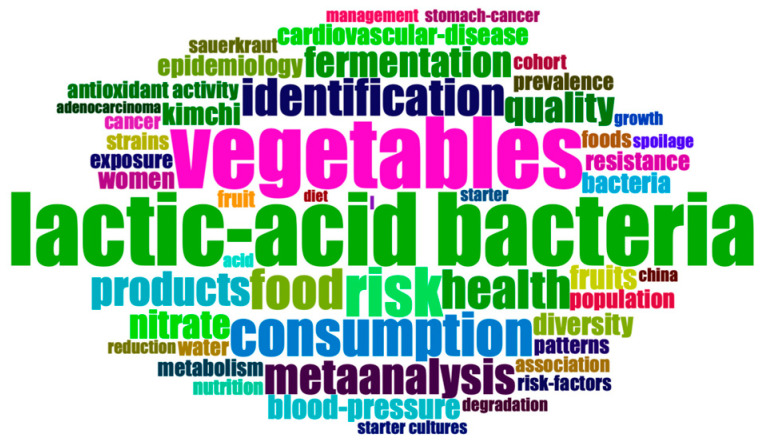
Word cloud analysis of current research on fermented vegetables.

However, consuming fermented vegetables still includes several health risks, such as high salt content, nitrite, biogenic amines, and pathogens and microorganisms. High salt intake is the cause of chronic diseases such as hypertension [33]. Nitrite has carcinogenic properties and is considered to be the primary cause of gastric cancer [34]. Biogenic amines are toxic and may cause allergic reactions in specific individuals [35]. In the meantime, fermented vegetables may be more prone to microbial contamination during the processing stage if the production process is not performed correctly [36]. Consuming fermented vegetables infected with pathogenic microorganisms poses a risk of foodborne pathogen infection since fermented vegetables are rarely exposed to thermal sterilization before being consumed. These adverse aspects make customers highly apprehensive about consuming fermented vegetables and hamper the development of fermented vegetables as functional foods [37]. For a long time, researchers have focused on exploring the bacterial community and flavor, and the relationships between them, in fermented vegetables. Several reviews on fermented vegetables have been published, focusing on the autochthonous microorganisms, bacterial community diversity [38], and the relationships between microbial diversity and the nutritional and health benefits of the pickles [39]. A word cloud analysis performed on 437 documents retrieved from the WOS core database using the keywords “ferment vegetable” and “fermented vegetable” over the past two decades [40] (Figure 1) shows that, in recent years, researchers have become increasingly interested in controlling risk factors and evaluating the health effects of fermented vegetables. However, few reviews have discussed the current understanding of the dynamic changes in the nutritional composition of fermented vegetables, their chemical and biological safety risks, and corresponding technological solutions.

Based on the current state of research, this article first summarizes the dynamic changes in nutritional components during the fermentation process of fermented vegetables, their health benefits after consumption, the current hazardous factors during the production of fermented vegetables, and new strategies and technologies for controlling these hazards, which would shed light on developing fermented vegetables as functional foods.

## 2. The Dynamic Process of the Fermentation of Pickled Vegetables

The fermentation of fermented vegetables is the consequence of microorganism metabolism based on the substrate of the raw materials and the added seasoning. Numerous complex biochemical reactions occur during the fermentation of fermented vegetables, involving microbial and nutritional composition changes.

### 2.1. Microbial Diversity in Fermented Vegetables

**Figure 2:**
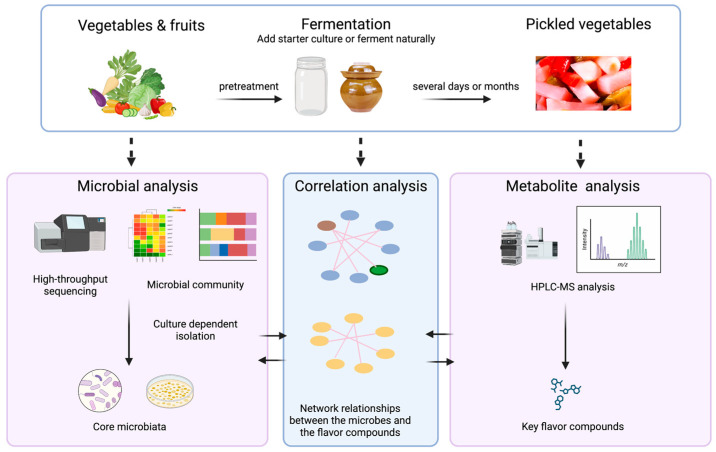
Schematic diagram of typical procedure for pickled vegetables production. Created with BioRender.com (https://www.biorender.com, accessed on 17 July 2023).

Multiple microbial communities interact within the complex microbial micro-environment of fermented vegetables. Lactic acid bacteria and *Bacillus* bacteria are two kinds of essential microorganisms with significant functions [41]. During the traditional fermentation process for fermented vegetables, their core microbial community is closely related to the ingredients, the place of the ingredient origin, the climate, the formula, the fermentation container, and the maker, which further affects the flavor of fermented vegetables [42,43]. The flavor is a critical indicator for determining the quality of fermented vegetables; therefore, by analyzing the correlation of the core microbiota that contributes to the fermented vegetables with excellent quality and flavor, and maintaining or improving the portion of these “good” microbes in the meantime, unfavorable microorganisms that deteriorate fermented vegetables can be controlled by appropriately manipulating the environmental parameters and other influence factors on purpose; implementing this within the industrial production of fermented vegetables with anticipated qualities [44]. Omics technologies, such as metagenomics and metabolomics, contribute significantly to investigating microbial communities and flavor compounds [45], as shown in Figure 2.

Studies have determined that the core microbes of various fermented vegetables are distinct; for instance, although the microbial flora in Sichuan paocai and spicy Chinese cabbage were dominated by Firmicutes and Proteobacteria, and both contain *Lactobacillus*, *Pediococcus*, *Serratia*, *Stenotrophomonas*, and *Weissella*, in paocai, *Stenotrophomonas* and *Serratia* were relatively abundant, while the amount of *Lactobacillus*, *Weissella*, and *Pediococcus8* in spicy Chinese cabbage was relatively high [46]. Similarly, the flavor and microbiota of fermented chili pepper and fermented radishes had similarities and distinctions [47]. 

Under the influence of certain bacteria and fungi, the compound related to the spicy flavor and raw taste, such as 3-butenyl isothiocyanate, 2-phenylethyl isothiocyanate, allyl isothiocyanate, 1-octen-3-ol, 3-hexen-1-ol 2-ethyl-1-hexanol, linalool, (E,E)-2,4-heptadienal and trans-.beta.-ionone, were partially or wholly decomposed after fermentation. Different substances predominate at various fermentation phases, resulting in flavor modifications. Twenty-five volatile compounds have been identified as having the highest impact on the aroma of fermented vegetables. The flavor is marginally dominated by bacterial influence. *Halanaerobium* and *Halomonas* are bacterial species with a minor advantage during the middle to late phases of fermentation. They can promote the production of butanoic acid and the breakdown of molecules like citric acid and malic acid, allyl isothiocyanate, 3-butenyl isothiocyanate, and 2-phenylethyl isothiocyanate [48]. Specific microorganisms can ferment insoluble substances in plants, such as xylooligosaccharides [49]. 

Seasonings added during fermented vegetable production may also influence fermented vegetables’ metabolites and microbial community alterations [50]. Adding wheat bran, for instance, can stimulate the proliferation of *Lactobacillus*, which can inhibit the growth of potential pathogens to some extent, and accelerate the fermentation process. However, due to the high nutrient content, the fermented vegetable also faces a risk of deterioration, necessitating a balance between the two [51]. Moreover, the influence of pesticide residue in raw materials on microbial diversity in fermented vegetables cannot be disregarded. The research found that no hetero lactic acid fermentation process was observed during fermentation, indicating that tetracycline has affected the metabolic pathways of lactic acid bacteria and the continuity of the LAB microbial community [52].

### 2.2. The Nutrition Composition Changes during the Fermentation Procedure of Fermented Vegetables

Marinating fresh vegetables and fruits alters their nutritional composition [53]. The fermentation process contributes to the loss or decomposition of essential nutrients (water, protein, fat, vitamin C, and phenolics) in vegetables by providing the energy required for the metabolic activity of the microbial flora [54]. However, at the same time, the metabolic activity of the strain can also produce new nutrient substances, thereby enhancing the nutritional value and probiotic effect of the fermented vegetables.

#### 2.2.1. The Dynamic Changes in the Primary Nutrients

In terms of the nutritional value of fermented vegetables, they will initially lose water and vitamin C during the pickling procedure. The water loss is because of the relatively high salinity of the fermented vegetables. Salt penetrates the raw material through pores formed on the surface in response to the change in external pressure, and this could be regarded as the primary and significant step in fermentation. The salt transport follows the law of fluid mechanics based on the diffusion of a concentration gradient. During this process, the water activity within the fermented vegetable materials changes. For the rigid or smooth surface skin of some vegetables lacking pores, salt diffusion is retarded, which might result in a more extended fermentation period that may require several months to obtain the pickles’ anticipated flavors [55]. The loss of ascorbic acid is because of the blanching procedure in hot water [15].

Other nutrient compositions also varied. The content of soluble protein decreased, but the content of polypeptides and free amino acids increased due to the effect of the protease secreted by certain protease-producing bacteria; the content of the umami, sweet, and bitter amino acids changes during the whole fermentation process, and the sweet amino acids (Thr, Ser, Gly, Ala, and Pro) would dominant as the fermentation continues [55]; in the meantime, the metabolic activity of strains such as *Lactobacillus* reduces the amount of anti-nutrient substances such as those that promote protein cross-linking and those that inhibit digestive enzymes; the presence of certain microorganisms can also promote the metabolic process in the human body, accelerate the decomposition of the toxins, and have the function of regulating and stabilizing the intestinal microenvironment. Consequently, the digestibility and bioavailability of the plant proteins in fermented vegetables are improved after consumption [56]. However, there are exceptions, such as fermented bamboo, whose soluble protein content would increase during fermentation (an increase from 3.1% to 7.8%) due to the proteolytic metabolic activities of the microorganisms, which make fermented bamboo a good source of digestible proteins [22]. For fat content, certain vegetables, such as broccoli, cucumber, and pepper, which are commonly used as pickling ingredients, may exhibit an increase in lipid content during pickling [57], which is also related to the enzymatic activities of fermenting organisms. In contrast, the lipid content in bamboo stalks decreases [22]. Fermentation alters the dietary fiber content of fermented vegetables by causing pectin disintegration in the cell wall and depolymerization under non-enzymatic action [58]; consequently, the texture of fermented vegetables will change [59].

#### 2.2.2. Generation of Other Nutritious Substances

The fermentation of fermented vegetables produces a variety of substances that are beneficial to the human body; fermentation significantly improves the nutritional value of leafy fermented foods [60]. Different types of fermented vegetables produce different types of active substances. Research has proved that the nutritional content in the leaves of *Amaranthus* sp. was improved after fermenting compared with the original content [61]. Cruciferae vegetables had the most significant variation in glucosinolates, polyphenols, and carotenoids. Most pickles have perfect antioxidant capacity due to the increase in the content of total phenols and flavonoids during fermentation [22]. Phenolic substances can inhibit xanthine oxidase by affecting the enzyme’s secondary structure and hydrophobic groups, thus controlling uric acid content in the human body [62]. 

Fermented vegetables such as kimchi produce various short-chain fatty acids, which would alter the structure of the host’s intestinal flora after intake [63]. Fermented cucumbers [24] produce GABA through the action of glutamate decarboxylase and arginine deiminase. In sauerkraut, glucosinolate breaks down into ascorbic acid and isothiocyanate [64], and in fermented olives, ascorbic acid and indole-3-carbinol were detected after marinating [65]. Fermented garlic produces more riboflavin and R-tocopherol than unfermented garlic [15]. For bamboo shoots, the p-cresol, α-gurjunene, methoxyphenyl oxime, and hexanal content could be retained; newly secreted substances included 1-nonanal, 4-methylanisole, β-cedrene, ethyl caprylate, (z)-2-heptanol, acetic acid, 1-hexanol, valeraldehyde, and ethyl palmitate, among which p-cresol has the highest content [66]. Fermented vegetables can also increase ions’ bioavailability due to the formation of Fe^3+^ [67].

The stability of these active substances will change during the storage of fermented vegetables. The GABA produced by fermented cucumbers remains stable for over six months of storage [23], while its total phenols’ stability depends on storage conditions. At the same time, the content of the ascorbic acid and isothiocyanate produced by sauerkraut [64], as well as the ascorbic acid and indole-3-carbinol produced by fermented olives [65], would decrease during storage. Therefore, to achieve the optimal probiotic effect of fermented vegetables, the best eating time for fermented vegetables is also a factor to be considered. However, some studies have found that long-term fermented vegetables may contain certain nutritional elements, such as β-sitosterol and its derivatives (β-sitosterol-3-O-glucose glycosides) which were isolated from 5-year-old fermented radishes, which have good binding properties with five antioxidant enzymes and have extraordinary antioxidant and bacteriostatic effects [68].

#### 2.2.3. Factors Affecting the Nutrient Changes in Fermented Vegetables

Changes in the nutritional components of fermented vegetables are influenced by various factors, including vegetable varieties, vegetable qualities, cultivation conditions, the addition of seasonings, pickling methods, and fermentation conditions [69]. Different flavors are imparted to fermented vegetables by different raw materials. For instance, the pigment, antioxidant properties, gluconapin, gluconasturiin, and total isothiocyanates would be distinct when different leaf mustard varieties were chosen when producing Guizhou sauerkraut [70]. Producing fermented vegetables using cabbage containing glucobrassicin could enhance the therapeutic effects of fermented vegetables on chronic diseases [71]. The phenolic acid content of traditional fermented bell peppers is more significant when sweet peppers are harvested using conventional cultivation methods. On the contrary, most flavonoids and carotenoids are found in organic samples [72]. The glutamic acid content of vegetables fermented with the addition of wheat bran increased significantly [73], and the levels of certain flavor compounds or components, such as free amino acids, α-linolenate, thiamine, and riboflavin, were increased. In addition, the level of sulfide compounds decreases, and the level of flavoring compounds rises, resulting in a significant reduction in the spicy flavor of fermented vegetables [74]. 

During the production of fermented vegetables, some operating units, such as blanching, affect the activity of alcohol acyltransferase (AAT) and would influence the content of myristicin and other quality parameters in nutmeg [75]. The fermentation conditions indirectly affect the nutrient changes in fermented vegetables. They would affect the nutritional composition of sauerkraut by interfering with the hydrolysis of volatile glucosinolates during fermentation [76]. The effect of the containers used in fermented vegetable production on their quality has also garnered considerable attention. The output of fermented vegetables in plastic containers has the fastest fermentation rate, and the concentrations of lactic acid and succinic acid were relatively high. In contrast, pickles in porcelain vessels contain more volatile compounds [77] and do not deteriorate quickly [78]. 

## 3. Health Benefits of Fermented Vegetables

The nutritional components generated during the pickling process and the probiotics in fermented vegetables are generally responsible for the health benefits of fermented vegetables. Table 1 summarizes some probiotics isolated from different fermented vegetables with distinct functions. 

**Figure 3:**
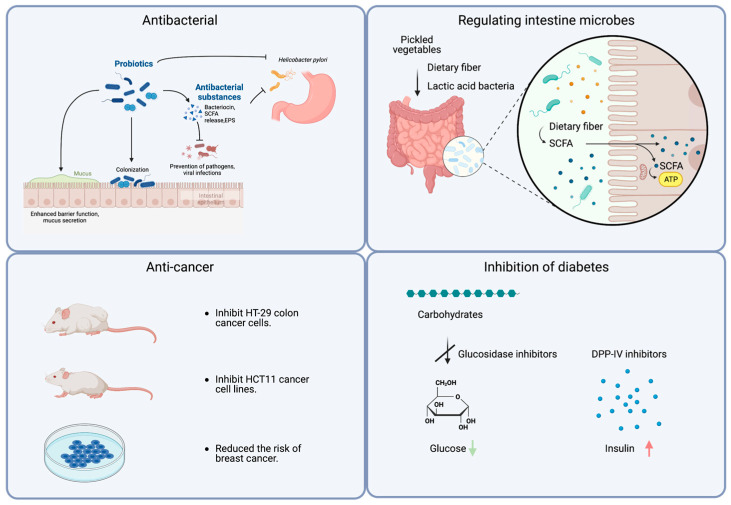
A schematic diagram illustrating the functions and potential mechanisms of pickled vegetables. Created with BioRender.com (https://www.biorender.com, accessed on 18 June 2023).

The health benefits of fermented vegetables include antibacterial effects, improvements in constipation, anticancer properties, the treatment of chronic diseases, the alleviation of irritable bowel syndrome, and immunity enhancement. Some of their main functions are shown in Figure 3.

### 3.1. Antibacterial

Due to the probiotics and their metabolites in fermented vegetables, such as antibacterial peptides and peroxides, one of the functions of fermented vegetables is the ability to inhibit the pathogen microorganisms. The primary mechanisms include disrupting cell structures, influencing the replication of genetic material, obstructing energy metabolism pathways, interfering with quorum sensing systems, regulating biofilm formation, and competing for essential nutrients. These antimicrobial substances include extracellular polysaccharides, phenolic compounds, and antibacterial peptides. *Helicobacter Pylori* (*H. pylori*) is considered the primary cause of stomach cancer, and eradicating this bacterium can be used as one of the therapies in its treatment. The research found that the extracellular polysaccharides produced by *Lactobacillus* sp. PW-7 isolated from fermented pickles can inhibit *H. pylori* [83]. Studies have shown that consuming kimchi can inhibit the growth of *H. pylori* in *H. pylori*-infected C57BL/6 mice [84]. Moreover, *Pediococcus pentosaceus* isolated from kimchi can inhibit *Listeria monocytogenes*, a Gram-positive bacterium that can readily cause listeria infection, and the antibacterial active site is on the LysM protein structure domain [85]. Probiotics in the pickles can generate a variety of antibacterial peptides, including those approved for use as food additives by the US Food and Drug Administration (FDA), such as nisin [86], and new antibacterial peptides are still being isolated from fermented vegetables [87]. In addition, a strain of *Lactiplantibacillus plantarum* CXG9 isolated from pickles can produce LD-phenylacetic acid [88]. Furthermore, phenolic compounds such as 2,6-dihydroxy acetophenone (DHAP), 4-hydroxybenzaldehyde (HBA), and 4-hydroxyphenyl alcohol (4-HPEA) have been discovered in sauerkraut juice and exhibit antibacterial activity to variable degrees [89].

### 3.2. Regulating the Intestine Microbes and Improving Intestine Health

Consuming fermented vegetables can increase intestinal microbiota diversity [90]. In addition to containing many lactic acid bacteria [27], most fermented vegetables are an essential source of dietary fiber and various vitamins. Moreover, they contain antioxidant-active compounds such as glutamine and glucosinolate. Black garlic, for instance, is abundant in short-chain fatty acids, which inhibit the development of harmful bacteria and promote the growth of beneficial bacteria. Oligosaccharides, such as fructooligosaccharides, melanoidins, and specific dietary phenolic substances, can also regulate intestinal microbiota [91]. The health effects of consuming fermented vegetables may also be long lasting [2]. Studies show that ingesting fermented vegetables for six months can ameliorate the imbalance in gut dysbiosis, and irritable bowel syndrome (IBS) symptoms can also be alleviated by consuming fermented vegetables fermented with lactic acid for some time [92]. Additionally, they can help to relieve constipation. Constipation diminishes the quality of life, and the accumulation of fecal contaminants in the intestines may increase the risk of intestinal diseases. In most cases, fermented vegetables and the probiotics they contain work together to treat constipation [93].

### 3.3. Anti-Cancer

The anti-cancer properties of fermented cabbage depend on the raw pickling ingredients. Studies have shown that fermented cruciferous vegetables have an anti-cancer effect, possibly related to the anti-cancer value of cruciferous vegetables being preserved after pickling based on in vitro experiments. A series of studies proved that kimchi could inhibit the proliferation of HT-29 colon cancer cells [94,95]. Studies showed that fermented mustard leaf does not affect normal colon myofibroblast CCD-18Co cells but can terminate the proliferation of HCT116 cancer cell lines and lead to large-scale cell apoptosis. Another study based on a 131 case-control study about breast cancer among Polish-born migrants in Cook County and the Detroit Metropolitan Area found that consuming fermented cruciferous vegetables can reduce the risk of breast cancer. According to their results, consuming raw or short-cooked fermented cabbage can substantially reduce the risk of breast cancer, while consuming long-cooked pickles has no association with it, possibly due to the probiotics or active substances being destructed by heat [96].

### 3.4. Inhibition of Diabetes

A disorder in carbohydrate metabolism primarily causes type 2 diabetes. Glucosidase inhibitors can delay the hydrolysis of carbohydrates into glucose, decreasing blood sugar levels. Inhibitors of dipeptidyl peptidase-IV (DPP-IV) prevent the degradation of glucagon-like peptide one (GLP-1) and gastric inhibitory peptide (GIP), thereby enhancing insulin secretion and lowering blood glucose levels since DPP-IV can substantially reduce GLP-1 and GIP; these two peptides can stimulate pancreatic insulin secretion after meals and significantly decrease blood glucose content. A ten-year prospective cohort study has confirmed that regular consumption of fermented vegetables reduces the risk of diabetes [97] because fermented vegetables contain luteolin and isorhamnetin-3-O-glucoside, a natural α-glucosidase and a potent inhibitor of DPP-IV, respectively [98]. 

## 4. Safety Problems in Fermented Vegetables and Current Solutions

**Figure 4:**
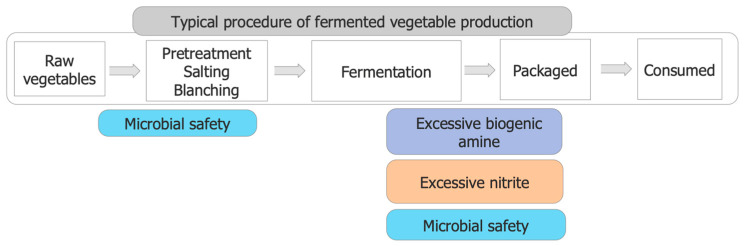
Safety problems occurred in different stages of fermented vegetable production.

Although there are nutritional and health benefits from fermented vegetables, their safety problems should not be neglected. Three main safety problems occurred in different stages of fermented vegetable production (Figure 4): biogenic amine, nitrite, and microbial safety.

### 4.1. Biogenic Amine

Biogenic amines result from the interaction of pickles’ ingredients and the metabolic activity of microorganisms [99]. The biogenic amine content of fermented vegetables is closely related to the biogenic amine content of the primary materials and the fermentation conditions [100]. Histamine, putrescine, and cadaverine are the primary biogenic amines found in fermented vegetables. Due to the difficulty of assuring hygienic conditions during fermentation, homemade fermented vegetables may contain more biogenic amines than commercially produced ones [101]. Reusing brine may also increase biogenic amine production [102]. The biogenic amine content of fermented vegetables from various regions may also vary [103].

The predominant beneficial bacterial species in fermented vegetables are *Lactobacilli*, particularly *L. plantarum*, and the presence of lactic acid bacteria can inhibit the proliferation of pathogenic bacteria to some extent; however, numerous studies have revealed that lactic acid bacteria can also produce biogenic amines [104], and for them, strain is more critical than species or genus in determining the biogenic amines-producing abilities of the lactic acid bacteria. Bacteria strains that can produce biogenic amines include *Enterococci*, *Lactobacilli*, *Streptococci*, *Pediococci*, and *Oenococci* [105]. *Lactobacilli* from several naturally fermented pickles can produce putrescine (PUT), cadaverine (CAD), and histamine (HIS) [106]. CAD and nitrite are associated with *Leuconostoc*, while *Lactobacillus* and *Pseudomonas* are associated with tyramine (TYR) [107]. *Lactobacillus brevis* primarily produces TYR [108]. Most studies, however, indicate that the levels of biogenic amines produced by these bacterial strains do not exceed the threshold for toxicity.

It is possible to reduce the biogenic amine content of fermented vegetables by manipulating the fermentation conditions. Although salt concentration has some effects on the formation of biogenic amines in certain varieties of pickles [109], studies have found that altering the salt concentration and temperature has a limited impact on inhibiting the formation of biogenic amines in pickles [110]. Changing the formula of pickles with a relatively low precursor of biogenic amines could, then, reduce its final content. For instance, adding fish sauce during the production of fermented cabbage can increase HIS; decreasing the additional amount of Myeolchi-aekjeot, a kind of fish sauce, can reduce the level of HIS and CAD in the final pickled products. Adding less fish sauce and more red pepper has the same effect on the total amount of biogenic amines content in kimchi [99]. Introducing onion and coriander can also reduce the concentration of biogenic amines. The addition of onion can inhibit four out of eight biogenic amines, including CAD, spermine (SPE), phenethylamine (PHE), and TYR, during the fermentation of sauerkraut, due to its antibacterial activity, which could inhibit the critical enzyme-producing bacteria in the biosynthesis of biogenic amines [111].

Selecting starter cultures of fermented vegetables that do not produce biogenic amines or can decompose biogenic amines is a highly effective strategy for reducing biogenic amines [108]. Various fermented foods have been discovered that contain bacteria capable of degrading biogenic amines [112], and the strains which are related to fermented vegetables are listed in Table 2. *Lactobacillus plantarum* [113], *L. plantarum* GZ-2, *L. brevis* SC-2 [107], *Levilactobacillus brevis* PK08, *Lactiplantibacillus pentosus* PK05, *Leuconostoc mesenteroides* YM20, *L. plantarum* KD15, and *Latilactobacillus sakei* YM21 [114] have all been identified as strains that do not produce or degrade biogenic amines. Different strains can degrade biogenic amines at varying rates [115]. *Staphylococcus carnosus* M43 can decompose HIS and TYR, while *Pediococcus acidilactici* M28 can decompose eight types of biogenic amines [116]. The biogenic degradation of the strains is firmly due to the secretion of biogenic amine-degrading enzymes [117]. *L. brevis* PK08, for instance, has a potent ability to degrade TYR, and this strain primarily degrades TYR by secreting multicopper oxidase (MCO) [114]. MCO can oxidize various phenolic and non-phenolic aromatic compounds while reducing dioxygen to water. Multicopper oxidase is superior to other biogenic amine-degrading enzymes since it has more potential applications [118]. In *Lactobacillus plantarum* J16 CECT 8944, another biogenic amine-degrading enzyme—laccase—was found, which has similar spectroscopic properties to blue copper oxidase and could primarily oxidize biogenic amines of the TYR type [119]. While another strain, *Halomonas shantousis* SWA25, can degrade a variety of biogenic amines. It can effectively degrade TRY, PHE, PUT, CAD, HIM, and TYR in fish sauce, and its biogenic amine-degrading action depends primarily on the membrane-distributed amine oxidase [120].

### 4.2. Nitrite

Nitrite, another unsafe substance in fermented vegetables, can form cancer-causing nitrosamines and induce cancer of the digestive system. The GB 2762-2017 standard (Food safety national standard food pollutant limits of China) specifies a maximum nitrite content of 4 mg/kg for raw vegetables and 20 mg/kg for fermented vegetables. The amount of nitrite in various pickles differs based on the ingredients used. According to recent studies, the maximum nitrite level has been found in fermented cabbage, followed by fermented mustard, bamboo, and radishes [122]. The concentration of nitrite is related to the composition of the microbial community of the pickles. The high-throughput sequencing results revealed an inverse relationship between the relative abundance of *Lactobacillus* and nitrite concentration [123], indicating that altering the microbial community’s structure by adding seasoning could reduce the nitrite concentration [123]. Studies have proved that adding garlic can substantially increase the number of *Lactobacillus* and *Weissella* in fermented vegetables and prevent the growth of undesirable microorganisms during fermentation. However, the nitrite residue was still relatively high in these pickles [124]. Another strategy for decomposing nitrite is by adding certain bioactive substances. Polyphenols extracted from apple rind with alkaline can effectively remove nitrite from pickles. Unlike ethanol-extracted polyphenols, non-ethanol-extracted polyphenols effectively eliminate nitrite without affecting the pickles’ flavor [125]. Furthermore, the addition of specific microelements, such as selenium, would accelerate the degradation of nitrite. Selenium (Se) can boost the antioxidant activity of lactic acid bacteria. The addition of Se can enhance the elimination ability of hydroxyl and superoxide radicals of the strain, enhancing the reaction rate of lipid peroxidation and ion-chelating and increasing the activity of superoxide dismutase (SOD) and glutathione peroxidase (GSH-Px), which are involved in the breakdown of nitrite [126].

Due to the growth of lactic acid bacteria in fermented vegetables during fermentation, nitrite can be degraded naturally [127]. Various strains that break down nitrite have been isolated from different pickles. These bacterial strains positively affect nitrite degradation when used as a starter culture for producing pickles and contribute to the excellent qualities of the fermented vegetables [128], including *Lactobacillus casei* subsp. *rhamnosus* LCR 6013 [129], *Lactiplantibacillus plantarum* ZJ316 [130], *Stachys sieboldii* Miq. [131], and *Lactobacillus coryniformis* [132]. There are currently three potential mechanisms which explain the nitrite-degrading abilities of the strains: acid degradation, enzyme degradation, and metabolic pathway degradation (Table 3).

Generally, nitrite decomposition can occur below a pH of 4 [133]. The low pH value results from the accumulation of organic acid produced by lactic acid bacteria, including lactic acid, acetic acid, butyric acid, tartaric acid, succinic acid, citric acid, and malic acid [131]. The nitrite content in mixed-strain fermented pickles is lower than in single-strain fermented ones because of the accumulation of organic acids. In addition, accumulating organic compounds can improve the flavor of pickles [134]. Hence, naturally fermented or mixed-strain fermented pickles would have great flavors.

Enzyme degradation is another mechanism used to reduce nitrite levels. Numerous lactic acid bacteria contain a nitrite reductase enzyme system that converts nitrite into NO_2_, NO, and N_2_ [123]. The nitrite reductase system of nitrite-reducing bacteria consists of genes such as *nirK*, *nirS*, and *nirBD* [135]. According to genomic research, nitrite-reducing bacteria have a certain tolerance for nitrite, and exposure to nitrite can cause elongation and shrinking in the bacteria, thereby decreasing their surface hydrophobicity. It was found that the genome of the strain contains genes encoding proteins and peptidoglycan proteins involved in regulating osmotic pressure, which can influence the expression of the cell wall in response to nitrite stress. In *L. plantarum* DMDL 9010, nitrite ions can bind to the active Cd1NiR (pgl) site through two hydrogen bonds [136]. There are also identical sequences of microbial nitrite reductase in other foods. For instance, mushroom-isolated nitrite reductase with a molecular weight of 90 kDa is homologous to the peptide sequence of fungi-derived nitrite reductase [137]. Consequently, some natural foods containing nitrite reductase have the potential to be used to produce fermented vegetables to reduce nitrite content.

A last nitrite degradation mechanism is metabolic pathway degradation [138]. A two-component system is capable of transferring nitrite to the pericytoplasm. The phosphotransferase system, glycolysis, and tricarboxylic acid cycle pathways generate reduced nicotinamide adenine dinucleotide (NADH) and flavin adenine dinucleotide-2 (FADH2). These substances produce electrons via the catalytic action of dehydrogenase catalytic reaction, and these electrons are transferred to nitrite via the electron transfer chain. Nitrite reductase reduces some nitrite molecules to NH_3_ by receiving electrons; glutamine synthetase then converts NH_3_ to L-glutamine [139]. In *Limosilactobacillus fermentum* RC4, its three secreted metabolites, mesaconate, 3-methylthiopropionic acid (MTP), and trans-aconitic acid, are effective at degrading nitrite. The particular mechanism is associated with the decarboxylation reaction [140], and it was found that *nirB* is associated with nitrogen metabolism [141]. There are also archaea capable of nitrite decomposition in fermented vegetables. Archaea obtain the electrons necessary for truncated denitrification by absorbing exogenous glucose from pickles, then they reduce nitrite to nitrogen with high efficiency, preventing nitrate from converting to nitrite [142]. *Halomicrobium* sp. ZPS, one of the nitrite-degrading archaea, has a mechanism for absorbing potassium and excluding sodium, and multiple varieties of nitrite reductase are involved in its nitrogen metabolism [143].

**Table 3:** Potential mechanisms behind the nitrite-degrading abilities of the microorganism.

Potential Mechanism	Factor That Play a Main Role	Main Results	Ref.
Acid degradation	The organic acid produced by the lactic acid bacteria	A low pH caused by the metabolic products (lactic acid, acetic acid, butyric acid, tartaric acid, succinic acid, citric acid, and malic acid) of the lactic acid bacteria, which cause the degradation of nitrite. Mixed strain fermentation has a more significant degrading effects.	[131,133,134]
Enzyme degradation	The nitrite reductase enzyme system exist in the microorganism	The nitrite reductase system of nitrite-reducing bacteria consists of genes such as *nirK*, *nirS*, and *nirBD*, which could convert nitrite into NO_2_, NO, and N_2_.	[123,135]
Metabolic pathway degradation	The particular metabolic pathway of the microorganisms	Received electrons generated by the glycolysis/gluconeogenesis and citrate cycle, which eventually convert nitrite to L-glutamine. Decarboxylation reaction. Denitrification.	[135,141]

### 4.3. Microbial Safety

During the production of fermented pickles, there are potential safety concerns associated with microbiological factors that could threaten consumers. It is necessary to control the pathogens at two time points; The first one is during the pre-treatment procedure, to reduce the miscellaneous bacteria, especially those that have strong biofilm-forming abilities; if these microorganisms are propagated and form a biofilm during the pickling procedure, the flavor of the fermented vegetables would be affected [144]. The other timepoint is at the end of the fermentation process. One purpose of this is to terminate the fermentation process, to avoid the adverse effect on the flavor and texture of the fermented vegetables by the excessive fermentation, but the most important purpose is to extend the shelf-life of the fermented vegetables and to maintain its edible safety for consumers; fermented vegetables were always considered to be ready-to-eat products in the market, for most of the homemade fermented vegetables, the sterilization in this step was always missing. Heat treatment is frequently used in the industrial sterilization of fermented vegetables at both time points. However, industrial sterilization alters the volatile compounds and texture of the pickles, hence decreasing consumer acceptability [145]. To address these issues, exploring novel non-thermal sterilization technologies to control harmful microorganisms arouses broad interest (Table 4). In the meantime, in conventional fermentation, a high sodium concentration is commonly used for microbial control; as mentioned above, the penetration of the salt to the fermented substrates depends on various factors, and a long period of salting could increase the risk of microbial deterioration; some non-thermal technologies can also accelerate the salting procedure.

#### 4.3.1. High-Pressure Processing (HPP) Technology

High-pressure processing (HPP) or high hydrostatic pressure processing (HHP) is a successful non-thermal commercial processing technology. In HPP processing, food is enclosed in flexible containers and subjected to 100–600 MPa of pressure at room temperature to achieve sterilization, with a liquid (usually water) as the pressure medium. As HPP does not alter low-energy covalent bonds, the primary structure of molecules (such as fatty acids and proteins) remains intact, whereas the secondary, tertiary, or quaternary structures of larger biological molecules such as membranes are disrupted by ionic bonds and hydrophobic interactions, resulting in changes in nutrient digestibility, bioavailability, and the technological and functional properties of the foods. However, molecules such as vitamins, amino acids, flavor compounds, and other substances with a low molecular weight are barely impacted [150]. After HPP treatment, the cells exhibit normal, sublethal status, which is reservable, and dead status owing to irreparable damage. For instance, research found that sublethal *E. coli* O157 can progressively recover to normal in PBS. High pressure affects the lipid component in the cell membrane by compressing the phospholipid bilayer and modifying cell fluidity, and the recovery process depends on temperature; the optimal temperature for recovery is 25 °C [151]. 

HPP treatment could inactivate the microbial populations during fermentation, inhibiting numerous spoilage microorganisms such as *Pseudomonas*, *Staphylococcus*, and *Shewanella* [152]. The sterilization in HPP depends on the pressure and the treatment time. HPP treatment towards pickled radish at 550 MPa for 5 min reduces total plate count (TPC) and substantially inactivates yeast and mold; similar to the sterilization results for thermal treatments, the pickles maintained microbial safety after sixty days of storage [146]. However, pressure treatment or storage following the pressure treatment may alter fermented vegetables’ nutritional components and the active substances. HPP treatment at 550 MPa for 5 min can increase the abundance of linalool, citronellol, and citral while decreasing the abundance of sulfide and terpinolene in fermented radishes, resulting in an increase in the sweetness and a decrease in the acrid odor of fermented radish. Thus, when implementing HPP treatment to control the hazardous microorganisms in the pickles, the optimal HPP parameters must be determined to minimize the impact on the quality of the pickles. 

For sauerkraut naturally fermented or fermented with starter cultures, HPP treatment diminishes the content of activated glucosinolates (AGB) by 33–67%, with no significant changes in the levels of indole-3-carbinol (I3C) and indole-3-acetonitrile (I3ACN); the reduction in vitamin C content of sauerkraut was significant [153]. HPP substantially affects the pH and color of fermented vegetables [154], can maintain the hardness to some extent [155], and has little effect on the microstructure of the pickle’s tissue [156]. Suppressing pectin esterification is the primary factor in maintaining the texture of the pickles [157]. HPP treatment and mixed-strain fermentation can increase fermented vegetables’ water-soluble dietary fiber and monosaccharide content. The consumption of dietary fiber can ameliorate the symptoms of many chronic diseases, including heart disease, obesity, type 2 diabetes, and colorectal cancer. After HPP treatment, more soluble dietary fiber can be extracted from the pickles [59]. At the same time, HPP combined with enzymatic hydrolysis can improve the functionality of insoluble dietary fiber (IDF) in fermented vegetables, as well as modify the IDF, including reducing their particle size, to form a loose and porous structure, improving their heat stability, and the alteration of the monosaccharide component, such as an increase in xylose and galactose contents. These modifications enhanced the absorption properties of the IDF for oil, glucose, nitrite, cholesterol, and Pb^2+^ [158], enhancing the nutritional value of fermented vegetables.

HPP technology could be used to replace the traditional thermal-and-soaking procedure, which might lead to the emergence of undesirable flavors. HPP can improve the myrosinase–glucosinolate system during fermentation; not only does HPP increase the migration rate of brines and disrupt the cell’s microstructure, but it also activates the myrosinase–glucosinolate system, resulting in a significant increase in the transformation of glucosinolate to isothiocyanate and then to sulforaphane. However, the activity of this enzyme system is relevant to the pressure level of HPP, and the activity of myrosinase can be inhibited at 600 MPa [152].

#### 4.3.2. Ultrasound Technology

Based on its frequency, ultrasound can be categorized as high-power–low-frequency (20–100 kHz), medium-power–medium-frequency (100 kHz–1 MHz), and low-power–high-frequency (1–100 MHz). Ultrasound can induce a cavitation effect, and the frequency used in food production is typically between 20 and 100 kHz [159]. Ultrasonic sterilization inhibits a wide range of microorganisms (bacteria, fungal, viruses), including *Escherichia coli*, *Staphylococcus aureus*, *Staphylococcus epidermidis*, *Enterobacter aerogenes*, *Bacillus subtilis*, *Aureobasidium pullulans*, hepatitis A virus, and murine norovirus [160,161,162]. The lethal effect of ultrasound on microorganisms depends on the species and morphology of the microorganisms. Gram-positive bacteria and yeasts with thicker cell membranes tolerate ultrasound relatively better than Gram-negative bacteria [160]. Microorganisms’ morphologies also influence their tolerance to ultrasound, with cocci exhibiting more extraordinary patience than bacilli due to their excellent surface area-to-volume ratio. For a particular microorganism, the stronger the ultrasonic treatment, the stronger the killing effect, and the possible anti-bacterial mechanism of high-intensity ultrasound might be significantly based on mechanical damage, generate greater free radical concentrations, destroy DNA, lipids and proteins required for cellular proliferation and metabolic activity [161].

Ultrasound sterilization is now used in microbial control in wine and juice but is rarely used in the sterilization of fermented vegetables, which might be due to the sterilization mechanism and the unique characteristics of ultrasound. However, ultrasound technology could overcome traditional methods’ limitations by accelerating salt dissolution and transport, facilitating the crucial fermentation step. Ultrasound treatment can maintain the firmness and texture of fermented cabbage, enhancing its palatability [163]. Additionally, ultrasound can speed up the fermentation process by improving the activities of β-glucosidase. β-glucosidase is a crucial enzyme in plant fermentation, which can convert glucoside flavonoids into aglycone glycoside flavonoids through hydrolyzing. According to research, the β-glucosidase activity in *Lactobacillus acidophilus* BCRC 1069 was doubled after treatment with 20 kHz ultrasound due to the cavitation effect induced by ultrasound, which increases the permeability of the cell membrane of the lactic acid bacteria, thereby facilitating the transport of β-glucosidase from the intracellular matrix to the extracellular matrix [164].

#### 4.3.3. Cold Plasma Technology

Plasma is an ionized gas composed of argon, helium, nitrogen, and compressed air. In an excited state, it produces charged particles, including electrons, ions, free radicals, and molecules [165]. Plasma can be generated using dielectric barrier discharge, corona discharge, micro hollow cathode discharge, or other discharge techniques. Two forms of plasma exist: thermal and non-thermal plasma, also known as cold atmospheric plasma. Compared to thermal plasma with a high temperature, CP has a lower operating temperature and, consequently, has minimal or no effects on food quality [166]. The anti-microbial effect of CP is primarily through an attack on cell structure and internal substances by ozone, charged particles, and oxygen radicals, which can destroy cell structure, damage internal components or DNA, and ultimately lead to cell mortality. Environmental factors such as pH, food matrix, relative humidity, and microbial species also affect the inhibitory effect of CP on foodborne pathogens. According to studies, CP has minimal effect on LAB and TVC but can selectively eradicate yeast. 

CP-treated fermented vegetables contain less soluble reducing sugar than pasteurized vegetables. The primary carbon source for microorganisms in fermented foods is the soluble reducing sugar, avoiding the growth of the gas-producing yeast, inhibiting peroxidase oxidation, regulating the nitrite content, and maintaining the color of the fermented vegetables, which, to some extent, avoids the excessive use of additives [147]. CP sterilization can enhance the qualities of some fermented vegetables [148]. For all we know, cold plasma is now rarely used in accelerating the salting process. 

#### 4.3.4. Photodynamic Sterilization 

Sterilization based on LED technology is an emerging technology used to control the microbial safety of food products. Blue light has a more significant sterilizing effect than red light [167]. Currently, ultraviolet-C light-emitting diodes control the white film formed on kimchi by inactivating the relevant yeast to regulate kimchi quality. According to research, LED treatment does not alter the physicochemical properties of kimchi, and the good quality of kimchi is maintained [149]. LED treatments also have numerous beneficial effects on the microbial community, metabolic rate, and functionality of kimchi [168]. 

#### 4.3.5. Pulsed Electric Field Technology (PEF) 

Pulsed electric field technology (PEF) is a method for treating food by applying short electric pulses (0–200 s) of high electric field intensity (1–10 KV/cm) to samples placed in an electric field chamber [169]. Like ultrasound sterilization, PEF is now used to inactivate the unanticipated microorganisms in other fields in the food industry, but it is rarely used to sterilize fermented vegetables. It was found that the PEF method can facilitate the seasoning fermented vegetables. Due to the electroporation effect caused by PEF altering the permeability of the cell membrane, the penetration of chemical substances into the cell was enhanced. It has been proven that this technique could be used to marinate fermented lotus roots. Food components, salt, and seasoning engage in intricate interactions during pickling. Water loss is a common occurrence during the pickling of vegetables and fruits. However, lotus root is rich in starch, which absorbs water and gelatinizes to create a gel layer on the sample’s surface, separating the internal cells from the brine. PEF treatment can permeate or dissolve this protective layer, thereby weakening its protective effect on the internal cells of the lotus root, accelerating the aggregation of NaCl in the sample, and decreasing the time required for NaCl to reach equilibrium. PEF can also soften fermented lotus root, lessen its chewiness, and enhance its flavor and appearance [170].

#### 4.3.6. How Far Are Non-Thermal Sterilization Technologies Being Applied in the Industry?

Currently, the application of non-thermal technologies is mainly focused on laboratory research. To ensure the widespread and successful adoption of non-thermal technology in industrial-scale production and its entry into the market, it is crucial to provide the necessary technical support and consider the non-technical elements, especially the psychological factors of consumers. Certain customers believed that the benefits of some novel technologies were exaggerated and preferred traditional techniques. Furthermore, a section of the consumers believe that the consumption of food processed using novel technology may potentially have adverse effects on their health. At the same time, the price is a critical determinant impacting consumer receptivity. Manufacturers must balance the cost and the prices of the products. Regional economic issues must also be considered when developing a sales plan. Consumers in less developed regions might prefer to emphasize the product’s price [171,172]. 

## 5. Conclusions

Researchers have accumulated significant knowledge about the core microbiota, key flavor compounds, and the relationships between the two in fermented vegetables. However, there is still a long way to go towards the booming industrial production of fermented vegetables. The interaction between the core microbiota, the substrates, and the flavor should be explored, and the underlying mechanisms between them must be elucidated to obtain uniform high quality across the fermented vegetables industry. More efficient technologies should be invented to guarantee the safety of fermented vegetables to avoid biogenic amines, nitrite, and pathogenic microorganisms. Considering the benefits and defects of fermented vegetables, more techniques are still needed to develop fermented vegetables as functional foods. One trend is to develop fermented vegetables based on plants from the ocean, such as seaweed, or fermented vegetables could be used as the ingredients in other products, like candy, dessert, ice cream, or bread, to invent a neo-functional food. 

In future investigations, the researchers could focus on the following directions: To consider the influence of fermented substrates, try to elucidate the interaction between the microbiota and the substrates precisely, especially when the fermentation was combined with novel technologies, like ultrasound, cold plasma, or the others mentioned in this review or not. Further elucidating the regulation mechanisms in the non-thermal technology used in the fermentation process and the growth and metabolite of core microbiota rather than inactivation them in the fermentation process, especially at the molecular level. The last is to develop novel fermented vegetables with the desired function that fully considers consumers’ acceptance. 

## Figures and Tables

**Table 1:** Probiotics in fermented vegetables and their function.

Fermented Vegetables	Probiotics	Function	Main Results	Ref.
Szechwan-style pickled vegetables	*Lactobacillus Plantarum* CQPC05	Inhibits constipation.	Up-regulated the mRNA expression of the stem cell factor receptor (c-Kit and SCF) and glial cell-derived neurotrophic factor genes, down-regulated the transient receptor potential cation channel subfamily V member 1 and inducible nitric oxide synthase.	[79]
Kimchi	*Lactococcus lactis* KC24	Antimicrobial, anti-inflammatory, antioxidant, anti-cancer.	*Listeria monocytogenes* and *Staphylococcus aureus* inhibition. Nitric oxide reduction. Inhibited gastric carcinoma (AGS), colon carcinoma (HT-29 and LoVo), breast carcinoma (MCF-7), and lung carcinoma (SK-MES-1) cells.	[25]
Kimchi	*Lactobacillus plantarum* EM	Lower cholesterol.	Cholesterol was removed by the cell wall fraction of the probiotics under the mechanism of enzymatic assimilation and was cell wall concentration-dependent.	[80]
Mango pickle	*Bacillus licheniformis* KT921419	Anti-cancer.	Works against the HT-29 colon cancer cell line	[29]
Chinese Sauerkraut	*Bacillus velezensis* T701	Antitumor.	The lipopeptide iturin A-2 produced by the strain showed good cytotoxic activities against Hela, MCF-7 and BT474 cell lines which related to cervical and breast cancer.	[81]
Sauerkraut	*Enterococcus*	Heavy metal elimination.	Eliminated heavy metals such as Cu, Pb, and Cd that are difficult to eliminate through cooking	[82]

**Table 2:** Starter cultures used in reducing the content of the biogenic amine in the fermented vegetable.

Strains	Isolation Origin	Characterization of the Strain and the Main Effects	Ref.
*Lactobacillus plantarum* GP11	Homemade pickled samples	Show no biogenic amine production ability. Exhibit antifungal activity against the *Aspergillus* sp. and *Penicillium* sp., which always leads to the contamination of the pickled vegetables.	[113]
*L. brevis* SC-2	Fermented mustard	A lower capacity of biogenic amine-producing ability, 13.95 mg/kg total biogenic amine producing ability with corresponding precursors; did not produce tryptamine, putrescine, and cadaverine, and could reduce the content of the biogenic amine in the fermented mustard from 137.16 mg/kg to 39.16 mg/kg	[107]
*L. plantarum* GZ-2	Fermented mustard	A lower capacity of biogenic amine-producing ability, 4.65 mg/kg total biogenic amine-producing ability with corresponding precursors, and could reduce the content of the biogenic amine of the fermented mustard.	[107]
*L. brevis* PK08	Kimchi	Has a multicopper oxidase gene, and showed a high reduction in tyramine content.	[114]
*Limosilactobacillus fermentum* G9	Cantonese pickles (containing mustard, cabbage, and bamboo shoots)	Has no biogenic amine-producing ability and could significantly reduce the biogenic amine content of Cantonese pickles to nearly 25 mg/kg compared to 150 mg/kg in the naturally fermented sample.	[121]

**Table 4:** Non-thermal technologies used in the sterilization of fermented vegetables.

Non-Thermal Technology	Sterilization Effects	Effects on the Sensory Quality of Fermented Vegetables	Ref.
HPP	HPP treatment at 550 MPa for 5 min reduces total plate count (TPC) and substantially inactivates yeast and mold in the pickled radish, and maintained microbial safety of pickles in sixty days of storage.	Might have adverse effects on the sensory quality of the pickled radish, and the treatment parameters should be prioritized.	[146]
	Maintained the shelf life of the marinated lotus root slices.	HPP treatment could retain the color and improve the flavor of the marinated lotus root slices.	[10]
Cold plasma	Could eliminated 5.00 logCFU/g of microorganisms under the CP treatment (voltage 60 kV, frequency 50 Hz, implementing time 60 s)	Increase the firmness of the radish paocai, could alleviate the softening and browning of radish paocai.	[147]
	Plasma activated water, generated by an AC bi-polar pulsed power supply (driving frequency 14.3 kHz, a peak-to-peak voltage 18 kV) for 120 s, could cause a reduction of 2.0, 2.2, 1.8, 0.9 log CFU/g mesophilic aerobic bacteria, lactic acid bacteria, yeast and moulds of ready-to-use shredded, salted kimchi.	Could reduce the salinity of peroxidase activity of the product.	[148]
Photodynamic	Could inhibit the while colony-forming yeast in kimchi seasoning	Maintain the volatile compounds in the kimchi seasoning	[149]

## Data Availability

Data is contained within the article.

## Abbreviations

GABA	γ-aminobutyric acid
LAB	lactic acid bacteria
Thr	threonine
Ser	serine
Gly	glycine
Ala	alanine
Pro	proline
DPP-IV	dipeptidyl peptidase-IV
GLP-1	glucagon-like peptide one
GIP	gastric inhibitory peptide
BA	biogenic amine
MCO	multicopper oxidase
PUT	putrescine
CAD	cadaverine
HIS	histamine
TYR	tyramine
SPE	spermine
PHE	phenethylamine
HPP	high-pressure processing
TPC	total plate count
IDF	insoluble dietary fiber
CP	cold plasma
PEF	pulsed electric field technology
